# CCR2/CCR5 Signaling Defines a Pathogenic Interstitial Macrophage Subset That Drives Severe Asthma

**DOI:** 10.1002/clt2.70201

**Published:** 2026-09-12

**Authors:** Hao Wang, Nok Him Fung, Christian Aloe, Nazanin Zounemat Kermani, Ian M. Adcock, Stavros Selemidis, Ross Vlahos, Jonathan McQualter, Steven Bozinovski

**Affiliations:** ^1^ School of Health and Biomedical Sciences RMIT University Melbourne Victoria Australia; ^2^ Centre for Respiratory Science & Health Melbourne Victoria Australia; ^3^ Imperial College London Data Science Institute London UK; ^4^ National Heart and Lung Institute (on behalf of the U‐BIOPRED consortia) Imperial College London London UK

## Abstract

**Background:**

Severe asthma is a chronic inflammatory disease characterised by airway dysfunction, persistent immune activation and corticosteroid resistance. While macrophages are key regulators of lung immunity, how individual subsets, particularly interstitial macrophages (IMs), contribute to disease remains poorly understood.

**Methods:**

Lung macrophages were profiled using single‐cell RNA‐sequencing (scRNA‐seq) and flow cytometry in a C57BL/6 mouse model of severe asthma induced by Complete Freund's Adjuvant (CFA) and house dust mite (HDM). Human transcriptomic data from the U‐BIOPRED asthma cohort were analyzed for translational validation. The dual CCR2/CCR5 antagonist Cenicriviroc (CVC) was used to evaluate the therapeutic potential of targeting pathogenic macrophages.

**Results:**

CFA‐HDM treatment led to marked depletion of resident alveolar macrophages (rAMs) and their replacement by recruited AMs (recAMs) associated with tissue remodeling. A distinct Lyve1^lo^MHCII^hi^ IM subset emerged as the dominant inflammatory cell population, displaying high *Ccr2* and *Ccr5* expression, potent pro‐inflammatory and antigen‐presenting signatures, and extensive ligand–receptor communication with T cells, neutrophils and rAMs. Trajectory analysis demonstrated that both recAMs and Lyve1^lo^MHCII^hi^ IMs arise from classical monocytes. Consistently, sputum transcriptomic data from the U‐BIOPRED asthma cohort showed reduced AM‐associated gene signatures and enrichment of IM/monocyte and CCR‐chemokine programs in patients with severe disease. Pharmacological blockade of CCR2/CCR5 with CVC markedly reduced IM infiltration, limited T‐cell and neutrophil recruitment, restored macrophage balance, and improved lung function.

**Conclusions:**

Lyve1^lo^MHCII^hi^ IMs orchestrate airway inflammation in severe asthma. Dual CCR2/CCR5 antagonism inhibits their recruitment, restores macrophage balance, and halts disease progression, highlighting this pathway as a promising therapeutic target.

## Graphical abstract

In mouse model of severe asthma, protective resident alveolar macrophages are depleted and replaced by monocyte‐derived populations, particularly a Lyve1^lo^MHCII^hi^ interstitial macrophage subset enriched for *Ccr2*/*Ccr5* and inflammatory gene programs. Blocking this subset with a dual CCR2/CCR5 antagonist reduces airway inflammation, restores homeostasis and attenuates AHR. A corresponding shift in macrophage transcriptomic programs is observed in patients with severe asthma. AHR, airway hyperresponsiveness; Alv. MΦ, alveolar macrophage; CCR2/CCR5, C‐C motif chemokine receptor 2/5; CFA, Complete Freund's Adjuvant; CVC, Cenicriviroc; ES, enrichment score; HDM, house dust mite; i.n. intranasal; Int. MΦ, interstitial macrophage; Mo, monocyte; s.c., subcutaneous; scRNA‐seq, single‐cell RNA‐sequencing; U‐BIOPRED, Unbiased Biomarkers for the Prediction of Respiratory Disease Outcomes.

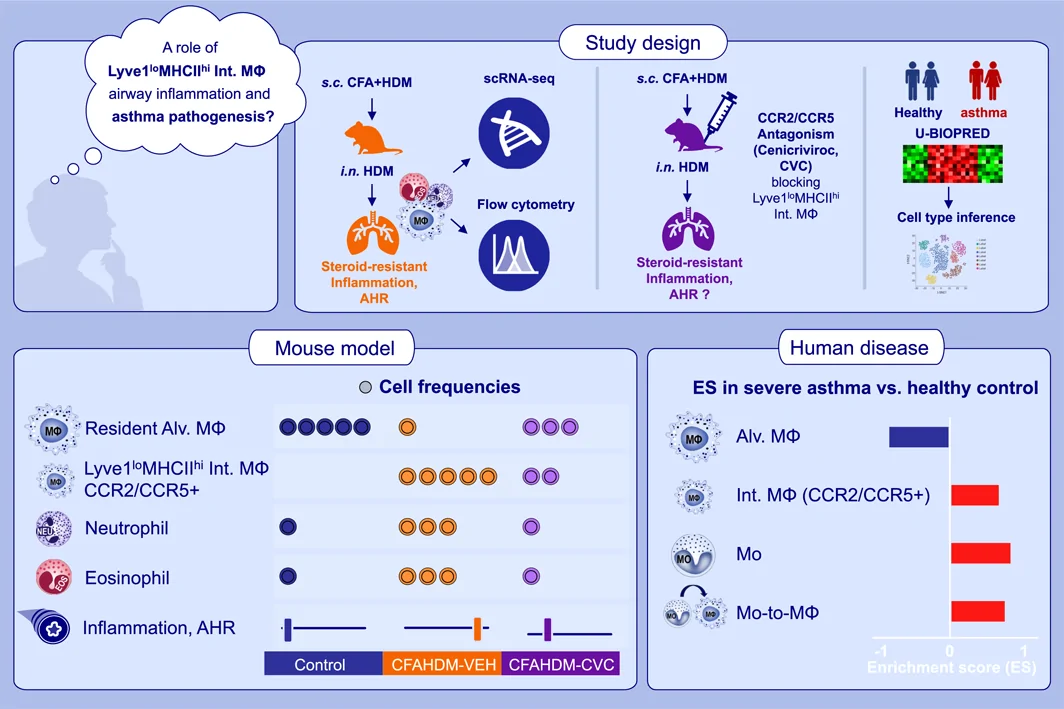

## 1 Introduction

Asthma is a chronic inflammatory lung disease characterised by airway hyperresponsiveness (AHR), airway remodeling, and persistent inflammation. Severe asthma represents a particularly debilitating phenotype, marked by resistance to corticosteroid therapy, progressive airway obstruction, poor disease control, frequent exacerbations, and a substantial healthcare burden [1, 2]. While traditionally associated with allergic, type 2 immunity, emerging evidence highlights macrophages as significant regulators of airway inflammation and tissue homeostasis [3, 4].

In healthy lungs, two distinct types of macrophages reside in specific tissue compartments: alveolar macrophages (AMs) in the airway/alveolar lumen and interstitial macrophages (IMs) in the lung interstitium/parenchyma [5]. AMs are primarily responsible for immune surveillance and maintaining homeostasis by catabolising alveolar surfactant and clearing cell debris, whereas IMs perform regulatory and tissue regenerative functions by producing the anti‐inflammatory cytokine IL‐10 and growth factors [6, 7, 8]. Unlike the relatively homogenous AMs [9], IMs are a heterogeneous population. Recent studies have identified at least two distinct subsets of conserved IM co‐exist at steady state: Lyve1^lo^MHCII^hi^ IMs, which are associated with inflammatory response and antigen presentation, and Lyve1^hi^MHCII^lo^ IMs, which are more involved in tissue repair and immunosuppression [10, 11, 12, 13, 14, 15, 16]. These IM subsets occupy different niches within the lung, with Lyve1^lo^MHCII^hi^ IMs localising around nerve bundles and Lyve1^hi^MHCII^lo^ IMs residing near blood vessels [10, 13]. Both AMs and IMs are considered tissue‐resident macrophages that are derived from yolk sac precursors or fetal liver monocytes during embryonic development [17]. While AMs are maintained by self‐renewal in adulthood, IMs are replenished by monocytes [10].

During inflammation, monocytes are recruited from blood or spleen to the lungs, giving rise to both AMs and IMs (referred to as recruited macrophages), which further adds to the complexity of the macrophage heterogeneity [16, 18, 19, 20]. In asthma, macrophage dysfunction has been linked to exacerbated airway inflammation, tissue remodeling and impaired host defences [21, 22]. However, it remains unclear whether these defects stem from intrinsic alterations in macrophage function or reflect shifts in macrophage subset composition during disease progression. Insights from murine models suggest that selective depletion of resident alveolar macrophages (rAMs) exacerbates allergen‐induced inflammation [23], whereas depletion of circulating monocytes prevents the recruitment of newly derived pulmonary macrophages and reduces Th2 cell‐mediated lung inflammation and remodeling [23, 24]. Recent finding suggests that IMs can effectively capture and present soluble antigens and trigger the proliferation of antigen‐specific CD4+ T cells [25]. Studies including our own, further demonstrate that targeting monocyte‐derived IMs either pharmacologically [26] or through genetic ablation [16] leads to improved airway pathology and lung function in the HDM model. Collectively, these findings suggest a protective role for rAMs and a pro‐inflammatory role for monocyte‐derived macrophages (mo‐Macs) in regulating allergic airway inflammation. Nonetheless, conflicting reports demonstrate that lung instillation of IL‐10‐producing IMs that were derived from blood or spleen monocytes prior to HDM challenge, protected mice from developing allergic airway disease [7, 19]. This discrepancy highlights the functional diversity of lung macrophages and underscores the need to distinguish pathogenic from protective IM subsets.

To address this, we employed single‐cell RNA sequencing (scRNA‐seq) and flow cytometry in a steroid‐resistant CFA‐HDM asthma model to dissect macrophage heterogeneity in airway inflammation. We identified a shift from rAMs toward monocyte‐derived macrophage subsets, with a prominent expansion of a pro‐inflammatory Lyve1^lo^MHCII^hi^ IM population expressing *Ccr2*/*Ccr5*. Transcriptomic analysis of patients with severe asthma in the Unbiased Biomarkers for the Prediction of Respiratory Disease Outcomes (U‐BIOPRED) cohort revealed a similar shift, supporting the relevance of these findings to human disease. Importantly, this Lyve1^lo^MHCII^hi^ IM subset was selectively targeted by the CCR2/CCR5 dual antagonist Cenicriviroc (CVC), which led to improvements in lung homeostasis and function. These findings identify a mechanistically distinct, CCR2/CCR5‐dependent macrophage population that drives severe asthma and establish IM‐directed chemokine receptor blockade as a promising therapeutic strategy.

## 2 Materials and Methods

### 2.1 Animals

All animal experiments conducted were approved by RMIT University Animal Ethics Committee (approval number: AEC26217) in accordance with ARRIVE guidelines. Female C57BL/6 mice (Ozgene‐ARC, Perth, Australia or WEHI, Melbourne, Australia) at 6–10 weeks of age were used in our studies.

### 2.2 Mouse Model of Steroid‐Resistant Asthma and CVC Treatment

Steroid‐resistant asthma was induced as previously described [27], by sensitising mice with subcutaneous injection of 100 μg of house dust mite extract (HDM) (*Dermatophagoides pteronyssinus*; Stallergenes Greer, US) in Complete Freund's Adjuvant (CFA, Sigma‐Aldrich, US), followed by an intranasal administration of 100 μg of HDM on day 14.

For the intervention study, we used a slightly modified protocol from our recent work [26], where mice were sensitised with subcutaneous injection of 100 μg of HDM in CFA and challenged 8 days later with intranasal HDM (25 μg) for two consecutive days. The modified protocol produced a comparable BAL and lung inflammatory cell profile while reducing the cumulative intranasal HDM dose and number of daily intraperitoneal injections, thereby reducing animal burden. Prophylactic treatment with CVC (30 mg/kg, MedChemExpress, US) or drug vehicle (VEH: 5% DMSO, 40%, 5% Tween‐80% and 50% saline; MedChemExpress, US) was administered daily via intraperitoneal injection throughout the sensitisation and challenge protocol [28]. Mice not exposed to CFA‐HDM and instead receiving intranasal saline (SAL) in place of HDM challenge were used as controls for all experiments, including scRNA‐seq and flow cytometry analyses.

Full descriptions of the methods are presented in the Supporting Information S1: Materials and Methods.

## 3 Results

### 3.1 Single‐Cell and Flow Cytometry Profiling Identifies Macrophage Subset Shifts in Severe Asthma

**FIGURE 1:**
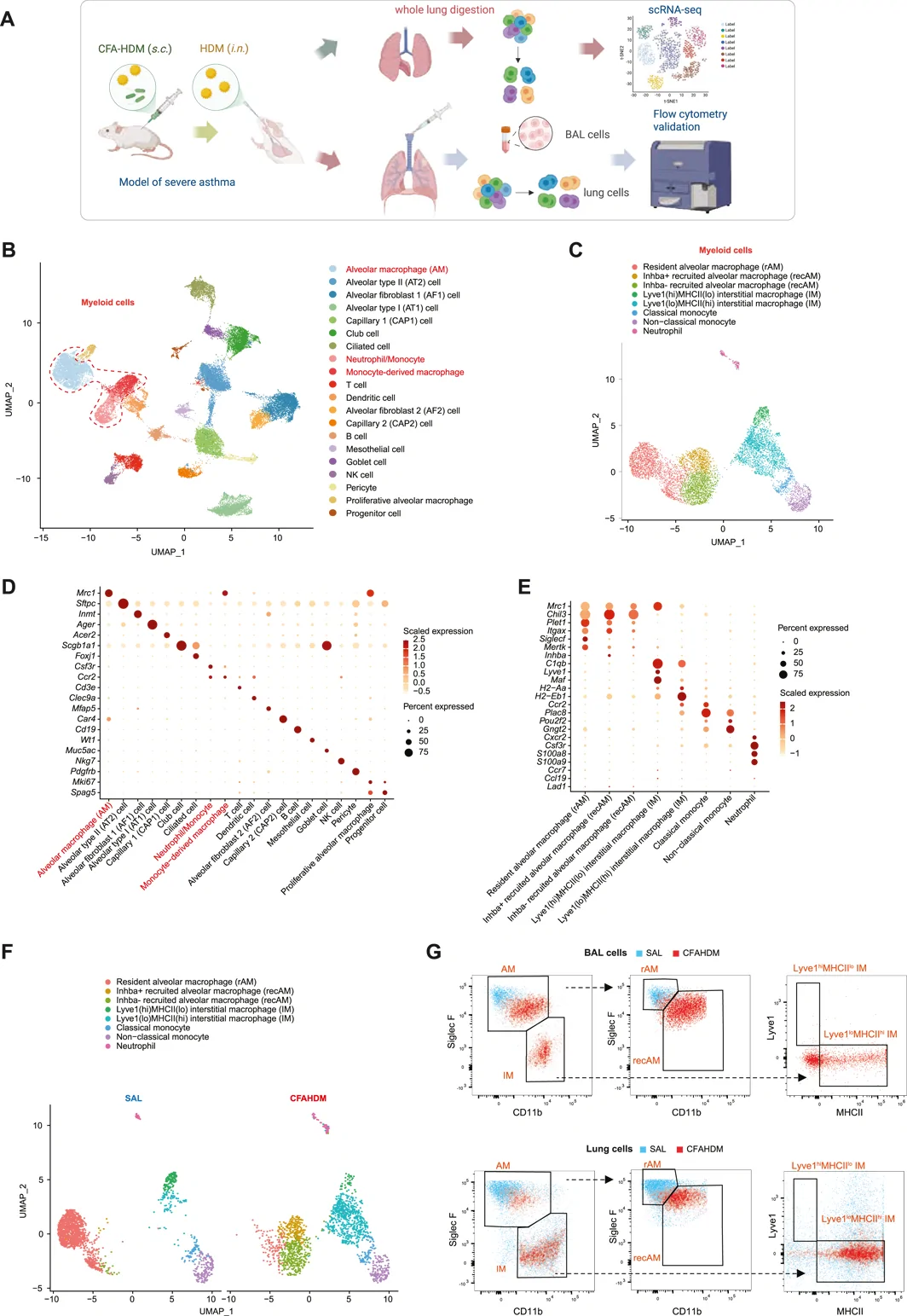
scRNA‐seq and flow cytometry identify transcriptionally and phenotypically distinct macrophage subsets in a mouse model of severe asthma. (A) Overview of the experimental workflow. A steroid‐resistant severe asthma model was established using CFA and HDM exposure. scRNA‐seq was performed on total lung cells from individual control (SAL) and CFA‐HDM‐treated mice (*n* = 3). Flow cytometry was performed on BAL cells and post‐lavage lung tissues from a separate cohort (*n* = 5–6). (B) UMAP plot displaying 20 cell types identified via *Louvain* clustering, with myeloid cells highlighted in red. (C) Sub‐clustering of myeloid cells further reveals 8 distinct subsets including neutrophils, monocytes, and macrophage populations. (D, E) Dot plots of representative marker genes used to define each major cell type (D) and myeloid subset (E). (F) UMAP visualisation of myeloid subsets across treatment groups, downsampled to equal cell numbers, highlights increased monocyte‐derived macrophages including recAMs and IMs, as well as depleted rAMs in CFA‐HDM exposed lungs. (G) Flow cytometry validation of rAM, recAM, and IM subsets in BAL cells (top) and lung tissue (bottom) confirms scRNA‐seq findings.

We first profiled the cellular landscape of the lung in our CFA‐HDM model of steroid‐resistant severe asthma [27] using scRNA‐seq on total lung cells obtained from unlavaged lungs, along with flow cytometry on BAL cells and post‐lavage lung tissue (Figure 1A). Using *Louvain* clustering, we first identified and annotated 20 cell clusters based on previously reported strategies [29, 30] (Figure 1B), with representative marker genes for each cluster shown in a dot plot (Figure 1D). To resolve the myeloid lineage, where clear distinction of neutrophils, monocytes and macrophage subsets were lacking, we conducted sub‐clustering on these cells. This further identified 8 distinct myeloid populations, including rAMs, recAMs (Inhba^+^ and Inhba^−^), IMs (Lyve1^lo^MHCII^hi^ and Lyve1^hi^MHCII^lo^), monocytes (classical and non‐classical), and neutrophils (Figure 1C). Corresponding marker genes are displayed in Figure 1E, and the mature dendritic cell markers *Ccr7*, *Ccl19*, and *Lad1* were confirmed to be absent in these cells. A more detailed comparison of macrophage and cDC marker gene expression across all cell types is provided in Supporting Information S1: Figure S1.

The recAM populations emerged exclusively in CFA‐HDM groups and were derived from the same parent AM cluster as rAMs. Although they retained canonical AM markers *Mrc1*, *Chil3* and *Plet1*, recAMs exhibited reduced *Siglecf* expression, consistent with transitional or monocyte‐derived AM phenotypes reported in chronic respiratory diseases and viral infection models [18]. These refined myeloid subsets were then re‐annotated in the main *Seurat* object, expanding the total number of identified cell types to 25 for downstream analyses. The proportional representation of each cell type is shown in Supporting Information S1: Figure S2A.

UMAP visualisation of the myeloid compartment revealed a profound reshaping of macrophage populations in severe asthma. rAMs were markedly depleted in the CFA‐HDM group, whereas Lyve1^lo^MHCII^hi^ IMs, neutrophils, and recAMs were substantially expanded (Figure 1F). These scRNA‐seq findings were validated using flow cytometry (Figure 1G). In BAL compartment (upper panel), two discrete macrophage populations (identified as CD45^+^Ly6G^−^MerTK^+^CD64^+^) were identified (gating strategy shown in Supporting Information S1: Figure S2B): AMs (SiglecF^hi^CD11b^lo^) and IMs (SiglecF^lo^CD11b^hi^). Within the AM gate, bona fide rAMs exhibited a SiglecF^bright^CD11b^−^ phenotype, whereas recAMs displayed reduced SiglecF expression and gained CD11b expression. Among IMs, the majority were Lyve1^−^MHCII^+^, with only a small fraction expressing Lyve1. In the SAL controls, only rAMs were detected, whereas CFA‐HDM exposed mice exhibited a near‐complete replacement of rAMs with recAMs and Lyve1^−^MHCII^+^ IMs. A similar shift in macrophage composition was observed in the lung tissue (Figure 1G lower panel & Supporting Information S1: Figure S2C).

### 3.2 Lyve1^lo^MHCII^hi^ IMs Exhibit Pro‐Inflammatory Signatures and Originate From Classical Monocytes

**FIGURE 2:**
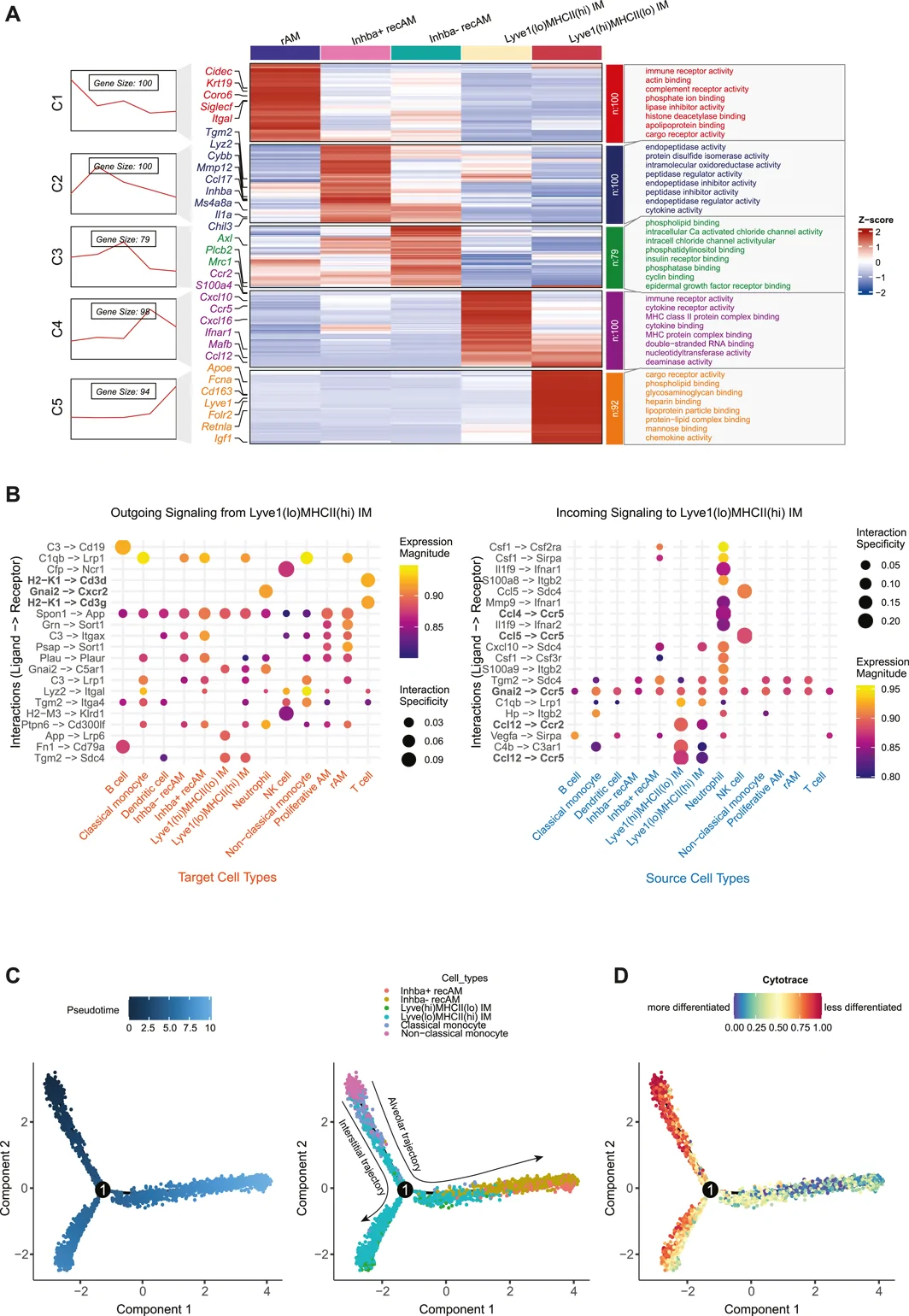
Lyve1^lo^MHCII^hi^ IMs exhibit pro‐inflammatory, interactive, and monocyte‐derived features in severe asthma. (A) Heatmap showing five transcriptional modules (C1–C5) derived from hierarchical clustering of differentially expressed genes across macrophage subsets. Each module is highlighted with representative genes and enriched Gene Ontology (GO) terms. The Lyve1^lo^MHCII^hi^ IM subset (yellow bar) is enriched in immune response, chemokine activity, and antigen presentation programs (in purple). (B) Cell‐cell communication analysis using *LIANA* reveals distinct ligand‐receptor pairs involving Lyve1^lo^MHCII^hi^ IMs. The left panel shows Lyve1^lo^MHCII^hi^ IMs as the source of outgoing signals to other immune cells, while the right panel shows all immune cells as the source and Lyve1^lo^MHCII^hi^ IMs as the target. Dot size indicates interaction specificity while color reflects expression magnitude. (C) *Monocle2* pseudotime trajectory analysis suggests a classical monocyte origin for Lyve1^lo^MHCII^hi^ IMs, with a developmental path branching into recAMs and IMs. Trajectory plots are colored by pseudotime (left) and cell identity (right). (D) *CytoTRACE* analysis confirms the pseudotime hierarchy inferred by *Monocle2*, supporting the developmental trajectory and monocyte‐derived nature of recAMs and IMs.

To define functional differences between macrophage subsets, we next performed differential gene expression analysis, followed by functional annotation of the top 100 differentially expressed genes (DEGs) in the Gene Ontology (GO) database (Figure 2A). The Lyve1^lo^MHCII^hi^ IMs expressed high levels of chemokine/cytokine and their receptor genes, including *Ccr2*, *Ccr5*, *Cxcl10*, *Cxcl16*, *Ifnar1* and *Ccl12*, indicating a role in inflammatory response, which was supported by enriched GO terms. Nucleotide modification and dsRNA binding, a process involving sterile activation of dsRNA sensors and implicated in chronic inflammation, were also enriched. In contrast, Lyve1^hi^MHCII^lo^ IMs expressed high level of M2 markers and growth factors, suggesting an involvement in immunosuppression and tissue repair. Pathways related to phagocytosis/efferocytosis (e.g., cargo receptor activity, mannose binding, and chemokine activity) and tissue remodeling/repair (e.g., glycosaminoglycan and heparin binding) were enriched in this cluster. Consistent with previous studies, Lyve1^hi^MHCII^lo^ IMs were identified as a source of IL‐10, while Lyve1^lo^MHCII^hi^ IMs displayed pro‐inflammatory and antigen‐presenting properties (Supporting Information S1: Figure S3A). Additionally, enriched GO terms in rAM suggest a role in phagocytosis, maintaining tissue homeostasis and regulating lipid metabolism, whereas recAM (Inhba^+^) were associated with proteolysis, ECM remodeling, and responses to oxidative stress.

To further delineate the intercellular communication networks involving Lyve1^lo^MHCII^hi^ IMs, we applied the *LIANA* and visualised the top 20 ligand‐receptor interactions in which Lyve1^lo^MHCII^hi^ IMs acted as either signal senders or receivers (Figure 2B). As signal senders (left panel), Lyve1^lo^MHCII^hi^ IMs established extensive communication with multiple immune cell types, underpinning their broad regulatory role. Notably, they appeared to facilitate antigen presentation to T cells via H2‐K1—Cd3d interactions, regulate neutrophil recruitment through Gnai2—Cxcr2 signaling, and reprogram rAMs through Grn/Psap—Sort1 and Plau—Plaur pathways. When functioning as signal receivers (right panel), Ccr2 and Ccr5 emerged as prominent receptors that mediate the recruitment and activation of Lyve1^lo^MHCII^hi^ IMs in severe asthma.

To explore the developmental relationship of the monocyte and macrophage subsets, we applied *Monocle 2*. It constructed developmental trajectories by modeling the gradual shifts in gene expression patterns as cells progressed through the differentiation process, and further conducted unsupervised ordering of these cells along the continuous trajectories in pseudotime. The analysis revealed two distinct developmental pathways (Figure 2C, left panel), with monocytes clustering at the root of the trajectory and representing the earliest pseudotime point. IM and recAM diverged into distinct terminal states with higher pseudotime values. Classical monocytes further showed a distribution pattern with cells densely packed at the beginning of the trajectory and progressively dispersed along both branches, which was observed at much lower frequency in non‐classical monocytes. This suggests that both the IM and recAM populations are primarily derived from classical monocytes. IMs were scattered along the trajectory whereas recAM were only found at the terminal branch, representing the most advanced differentiation status. Consistently, a heatmap of genes co‐varied across pseudotime revealed high expression of classical monocyte‐associated genes, such as *Tmsb4x*, *Plac8*, and *Fyb* in the pre‐branch state, with expression gradually declining as cells progressed toward either fate (Supporting Information S1: Figure S3B). In contrast, IM‐enriched genes (e.g., *C1qb* and *Marcks*) and AM‐enriched genes (e.g., *Chil3* and *Ctsk*) were initially low in the pre‐branch state and increased as cells transitioned toward their respective IM or AM identities. Transcriptional factors *Maf* and *Mafb* that were previously identified to regulate IM differentiation were also present. These findings were independently assessed by *Cytotrace*, an algorithm infers the differentiation potential of cells by using the number of detectably expressed genes per cell as a surrogate, which showed identical results as *Monocle 2* (Figure 2D).

### 3.3 Human Transcriptomic Profiling Using U‐BIOPRED Data Confirms Macrophage Subset Reprogramming in Severe Asthma

**FIGURE 3:**
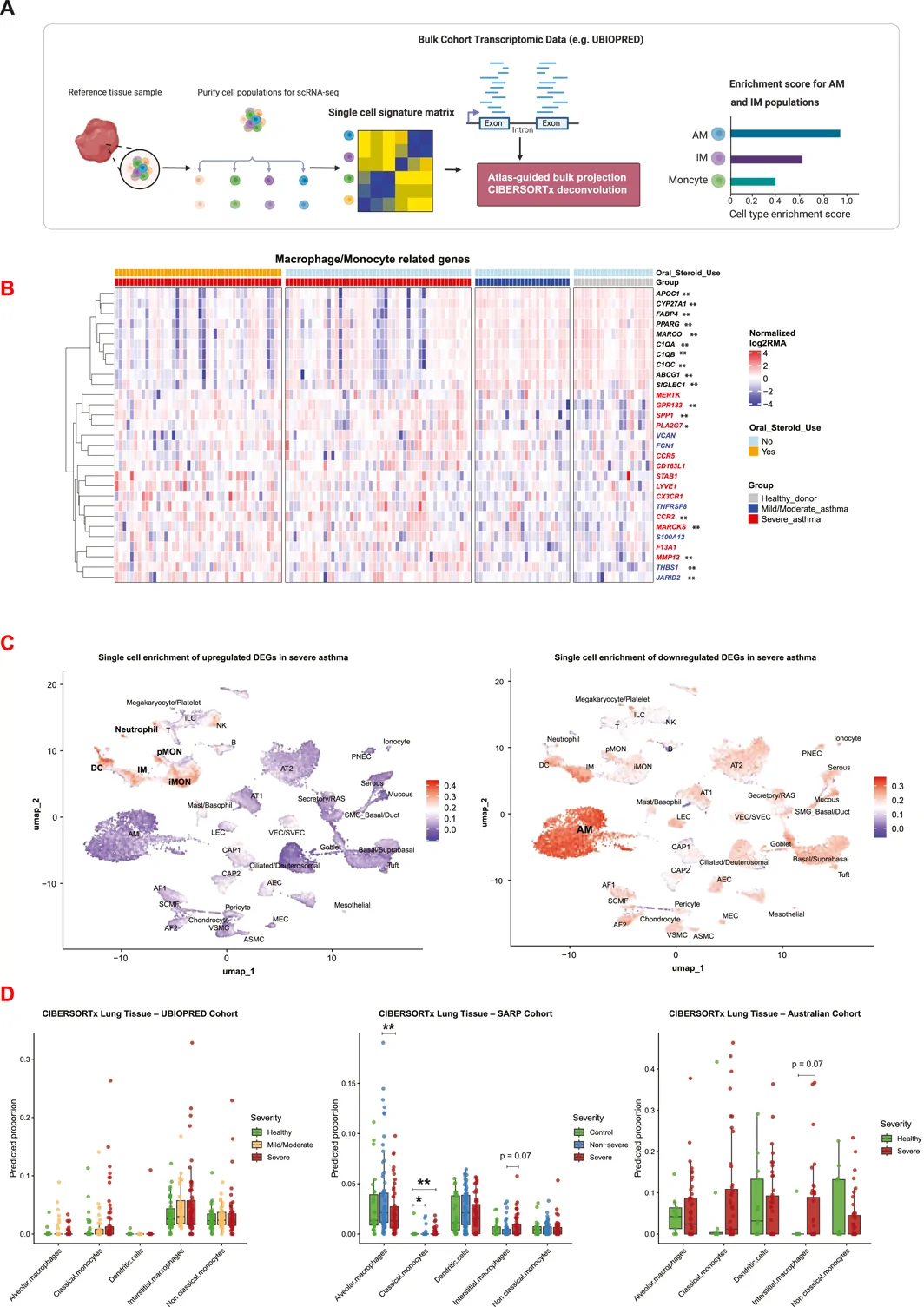
Enrichment of IM and monocyte signatures in patients with severe asthma. (A) Schematic overview of the in silico deconvolution workflow. Sputum transcriptomic data from the U‐BIOPRED cohort, consisting of 21 healthy control, 25 patients with moderate asthma and 93 patients with severe asthma were analyzed. (B) Heatmap showing the expression of macrophage/monocyte‐related genes across healthy controls, moderate asthma, and severe asthma groups. Orange/light blue annotations indicate oral steroid use. Gene labels in black indicate AM‐associated genes, labels in red indicate IM‐associated genes, and labels in blue indicate classical monocyte‐associated genes. ** adjusted *p* < 0.05 and * *p* < 0.05 for severe asthma versus healthy controls using *Limma*. (C) Differentially expressed genes (DEGs) including both upregulated (left) and downregulated (right) genes in severe asthma were projected on to a human lung single‐cell atlas established by the LungMAP consortium. Enrichment scores were calculated at the single‐cell level. (D) Cellular deconvolution of epithelial brushing and bronchial biopsy transcriptomes from three independent severe asthma cohorts—U‐BIOPRED, SARP (GSE63142), and an Australian cohort (GSE143303), was performed using an established airway single‐cell reference and analytical pipeline from the Human Lung Cell Atlas (HLCA). Only myeloid cell types are shown here, with the full list of cell types presented in Supporting Information S1: Figure S5. Data are presented as median with interquartile range (IQR). *p < 0.05, **p < 0.01, ***p < 0.001, ****p < 0.0001 by Mann–Whitney *U* test or the Kruskal–Wallis test with Dunn's multiple comparisons.

To determine whether the macrophage shifts observed in mice were conserved in humans, we performed transcriptomic profiling of sputum samples from the U‐BIOPRED cohort as these samples are enriched in macrophages (Figure 3A). Across healthy controls, moderate asthma, and severe asthma subjects, genes associated with mature AMs (e.g., *MARCO*, *FABP4*, *MRC1*) were significantly reduced in severe disease (Figure 3B). In contrast, genes linked to IMs (red) and classic monocytes (blue), including *GPR183*, *SPP1*, *MARCKS*, *MMP12*, *CCR2*, *THBS1*, and *JARID2*, were significantly upregulated, although *CCR5* showed only a trend of increase.

Projection of these differentially expressed genes (DEGs) onto a human lung single‐cell atlas (LungMap) revealed a marked enrichment of upregulated genes in myeloid populations, including classical monocytes and IMs, whereas downregulated genes were predominantly enriched in AMs (Figure 3C). GSVA across individual samples (Supporting Information S1: Figure S4A) supported these observations, which revealed significantly reduced enrichment scores (ES) for AMs and elevated ES for IMs and classical monocytes in severe asthma, but not in mild‐to‐moderate disease, relative to healthy controls. In addition, *CCR2‐CCR5* ES also trended toward an increase in severe asthma (*p* = 0.08 vs. healthy controls). These transcriptional shifts were accompanied by the enrichment of functional gene sets associated with monocyte chemotaxis, monocyte differentiation, macrophage differentiation, and CCR receptor binding (Supporting Information S1: Figure S4B).

As no validated single‐cell reference matrix is currently available for direct sputum deconvolution, we instead analyzed epithelial brushing and bronchial biopsy transcriptomes from three independent severe asthma cohorts [31, 32, 33] using an established airway single‐cell reference and pipeline by Human Lung Cell Atlas (HLCA) [34] (Supporting Information S1: Figure S5). While immune cells, including IMs, are present at relatively low abundance in these tissue compartments, both IMs and classical monocytes were detected at a higher frequency in severe disease. Consistently, predicted IM and classical monocyte proportions tended to increase in severe disease, whereas AM proportions showed a decreasing trend (Figure 3D). Collectively, these findings suggest a reshaping of the airway myeloid compartment in severe asthma, reflected by a loss of rAM programs and enrichment of IM‐ and monocyte‐related signatures.

### 3.4 CCR2/CCR5 Dual Antagonism Attenuates Airway Inflammation and AHR In Vivo

Since the Lyve1^lo^MHCII^hi^ IMs expressed high levels of *Ccr2* and *Ccr5*, we next sought to investigate whether pharmacological inhibition of these receptors could suppress their accumulation and improve lung function. Under non‐disease conditions, *Ccr2‐Ccr5* enrichment was largely restricted to myeloid cells and T cells across both human and mouse reference single‐cell atlases (Supporting Information S1: Figure S6A,B), a pattern also observed in SAL‐treated mice in our study (Supporting Information S1: Figure S6C). In contrast, in CFAHDM‐exposed mice, *Ccr2‐Ccr5* enrichment was predominantly observed in Lyve1^lo^MHCII^hi^ IMs (Supporting Information S1: Figure S6D). This finding was further validated at the protein level by flow cytometry, which demonstrated robust co‐expression of CCR2 and CCR5 within this IM subset in CFAHDM‐exposed mice (Supporting Information S1: Figure S7).

**FIGURE 4:**
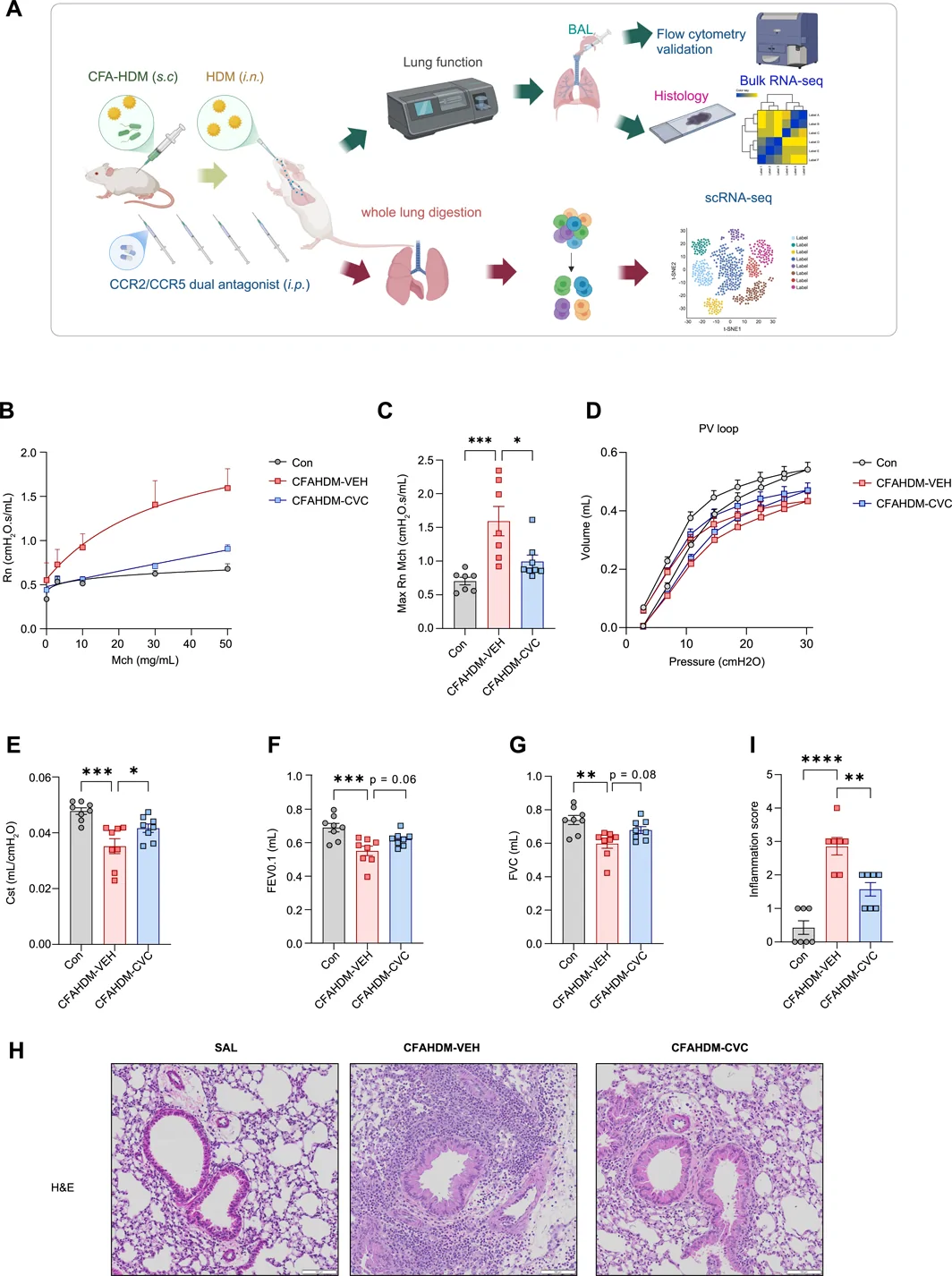
CCR2/CCR5 antagonism with CVC prevents AHR and improves lung pathology in CFA‐HDM induced asthma model. (A) Schematic of the experimental workflow. Mice were sensitised subcutaneously with CFA‐HDM and challenged intranasally with HDM to induce steroid‐resistant severe asthma. Cenicriviroc (CVC), a dual CCR2/CCR5 antagonist, or vehicle control (VEH) was administered daily throughout the protocol. scRNA‐seq was performed on total lung cells from three mice per group (CVC and VEH). A separate cohort of mice were assessed for baseline lung function, including pressure‐volume (PV) loops, lung volumes, and airway resistance (Rn) in response to methacholine (MCh) challenge. Lung samples were processed for BAL, flow cytometry, histology, and bulk RNA‐seq. (B) Airway resistance in response to increasing MCh doses. (C) Maximal airway resistance detected at the highest MCh dose. (D) PV loop curves showing a downward shift in CFA‐HDM‐VEH mice and partial reversal with CVC treatment. (E) Quasi‐static lung compliance (Cst) derived from PV loops. (F, G) Lung volumes including FEV0.1 (F) and FVC (G) measured through negative pressure‐forced expiratory (NPFE) maneuvers. (H) Representative H&E‐stained lung sections showing tissue inflammation. (I) Quantification of lung inflammation scores. Data are presented as mean ± SEM. *n* = 7–8 per group. *p < 0.05, **p < 0.01, ***p < 0.001, ****p < 0.0001 by one‐way ANOVA.

Subsequently, CFA‐HDM exposed mice were treated daily with Cenicriviroc (CVC), a clinically tested dual CCR2/CCR5 antagonist, or vehicle control (VEH) throughout the sensitization and challenge period (Figure 4A). CVC completely prevented CFA‐HDM‐induced AHR in response to methacholine (Mch) challenge, as measured by the reduction in airway resistance (Rn) in CVC‐treated mice compared to VEH‐treated mice (Figure 4B,C). Airway restriction, reflected by the downward shift in the pressure‐volume (PV) loop curve (Figure 4D), reduction in the quasi‐static compliance (Cst) (Figure 4E), and reduction in lung volumes FEV0.1 (Figure 4F) and FVC (Figure 4G) were reversed by CCR2/CCR5 dual antagonism. At the histopathological level, CFA‐HDM induced pulmonary inflammation determined by H&E staining (Figure 4H) was attenuated by CVC (Figure 4I). These findings demonstrate that CCR2/CCR5 blockade effectively preserves lung mechanics, reduces airway inflammation, and prevents the development of steroid‐resistant AHR.

### 3.5 CVC Treatment Reverses Inflammatory Cell Expansion and Restores Epithelial Balance

**FIGURE 5:**
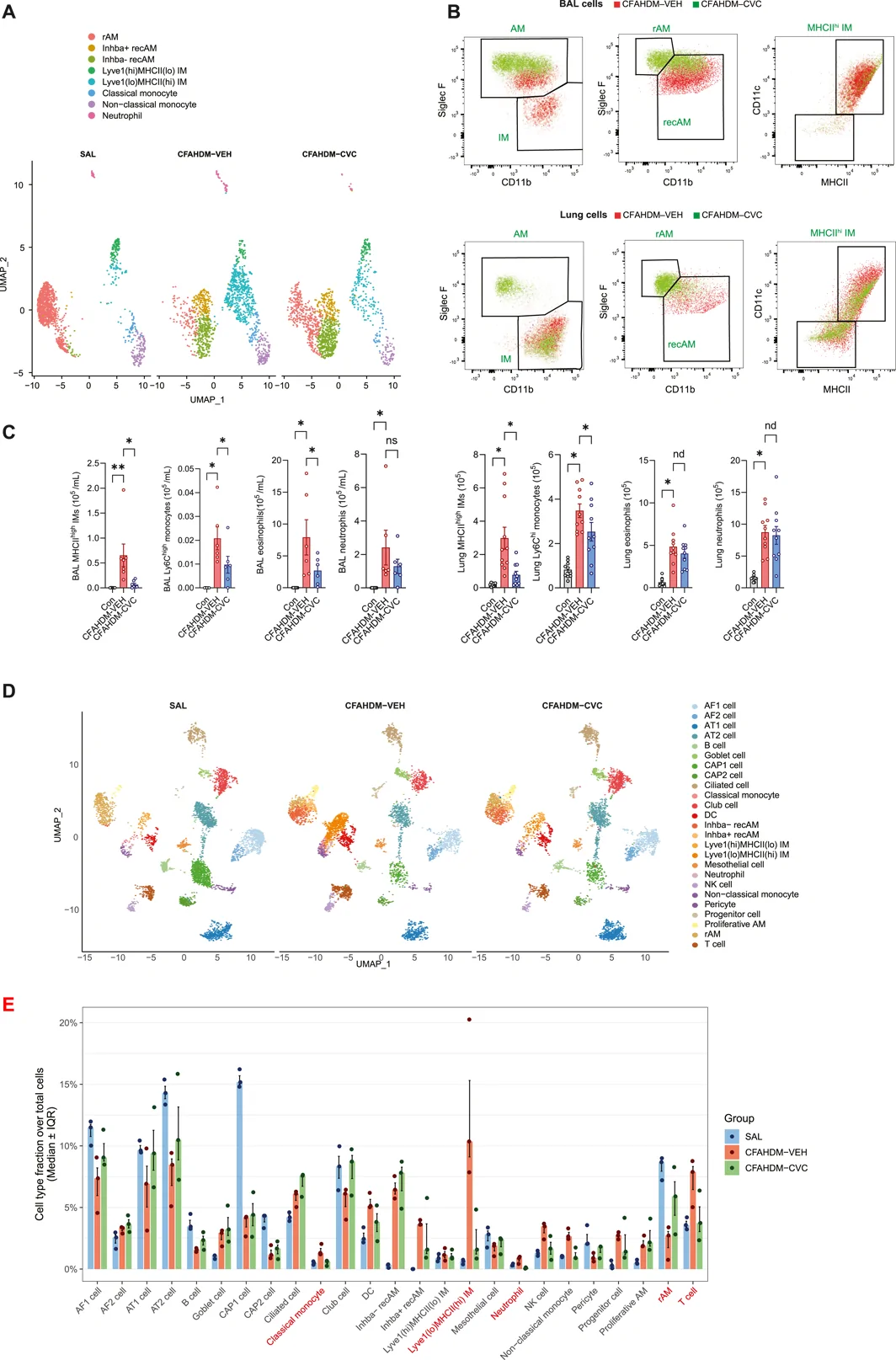
CCR2/CCR5 antagonism with CVC restores alveolar macrophage populations and reduces inflammatory cell infiltration in CFA‐HDM exposed lungs. (A) UMAP visualisation of myeloid cell clusters derived from scRNA‐seq data, downsampled to equal cell numbers across SAL, CFA‐HDM‐VEH, and CFA‐HDM‐CVC groups (*n* = 3). CFA‐HDM‐VEH mice showed a marked expansion of monocyte‐derived macrophages including recAM and IM, as well as depletion of rAMs, all of which were largely prevented by CVC treatment. (B) Flow cytometry validation of rAM, recAM, and IM subsets in BAL cells (top) and lung tissue (bottom) confirms scRNA‐seq findings. (C) Quantification of flow cytometry results confirms the reduction of MHCII^hi^ IMs in the BAL and lung tissue, and further reveals reduction in BAL eosinophils, and neutrophils (*n* = 7–8). Data are presented as mean ± SEM. *p < 0.05, **p < 0.01 by one‐way ANOVA. (D) UMAP of visualisation of all lung cells from scRNA‐seq data, downsampled to equal cell numbers across SAL, CFA‐HDM‐VEH, and CFA‐HDM‐CVC groups. Cells are colored by annotated cell types, including immune and structural cell populations. (E) Bar plots showing the fraction of each annotated cell type over total cell number, highlighting immune cell population shifts and lung remodeling following CFA‐HDM exposure and the partial restoration by CVC treatment. Data are presented as median with interquartile range (IQR).

In the scRNA‐seq analysis of the myeloid cells, CVC treatment prevented the Lyve1^lo^MHCII^hi^ IM expansion induced by CFA‐HDM, whereas Lyve1^hi^MHCII^lo^ IMs were not affected (Figure 5A). Classical monocytes and neutrophils, both elevated in the CFA‐HDM‐VEH group, were also significantly reduced with CVC treatment, and the depletion of rAMs was partially rescued. These scRNA‐seq findings were validated by flow cytometry, which confirmed a significant reduction of MHCII^high^ IMs in both BAL and lung compartments following CVC treatment (Figure 5B,C). CVC also partially restored the rAM population and decreased BAL eosinophils and neutrophils (Figure 5C), consistent with an overall attenuation of airway inflammation.

Beyond the myeloid compartment, global changes in the cellular landscape were visualised in the UMAP across the three experimental groups (Figure 5D). Quantitative analysis of immune populations revealed that T cells and NK cells, elevated in CFA‐HDM‐VEH, were reduced by CVC, whereas DC numbers remained unchanged (Figure 5E). Within the T cell compartment, both Th1 and Th2 cells were expanded in CFA‐HDM‐VEH and reduced by CVC (Supporting Information S1: Figure S8A). Consistently, CFA‐HDM‐induced changes in myeloid‐associated chemokine signaling, T cell‐associated transcriptional programs, macrophage subset markers, and inflammatory mediators in CFA‐HDM‐VEH were partially prevented by CVC treatment (Supporting Information S1: Figure S8B). Alterations to the non‐immune cells by CFA‐HDM exposure were also partially reversed following CVC treatment (Figure 5E). Collectively, these data demonstrate that CVC treatment not only reprograms inflammatory myeloid populations but also contributes to the restoration of epithelial homeostasis in the inflamed lung, ultimately ameliorating key steroid‐resistant features of the disease including AHR.

### 3.6 CVC Reprograms Macrophage Transcriptomes From Inflammatory to Reparative States

**FIGURE 6:**
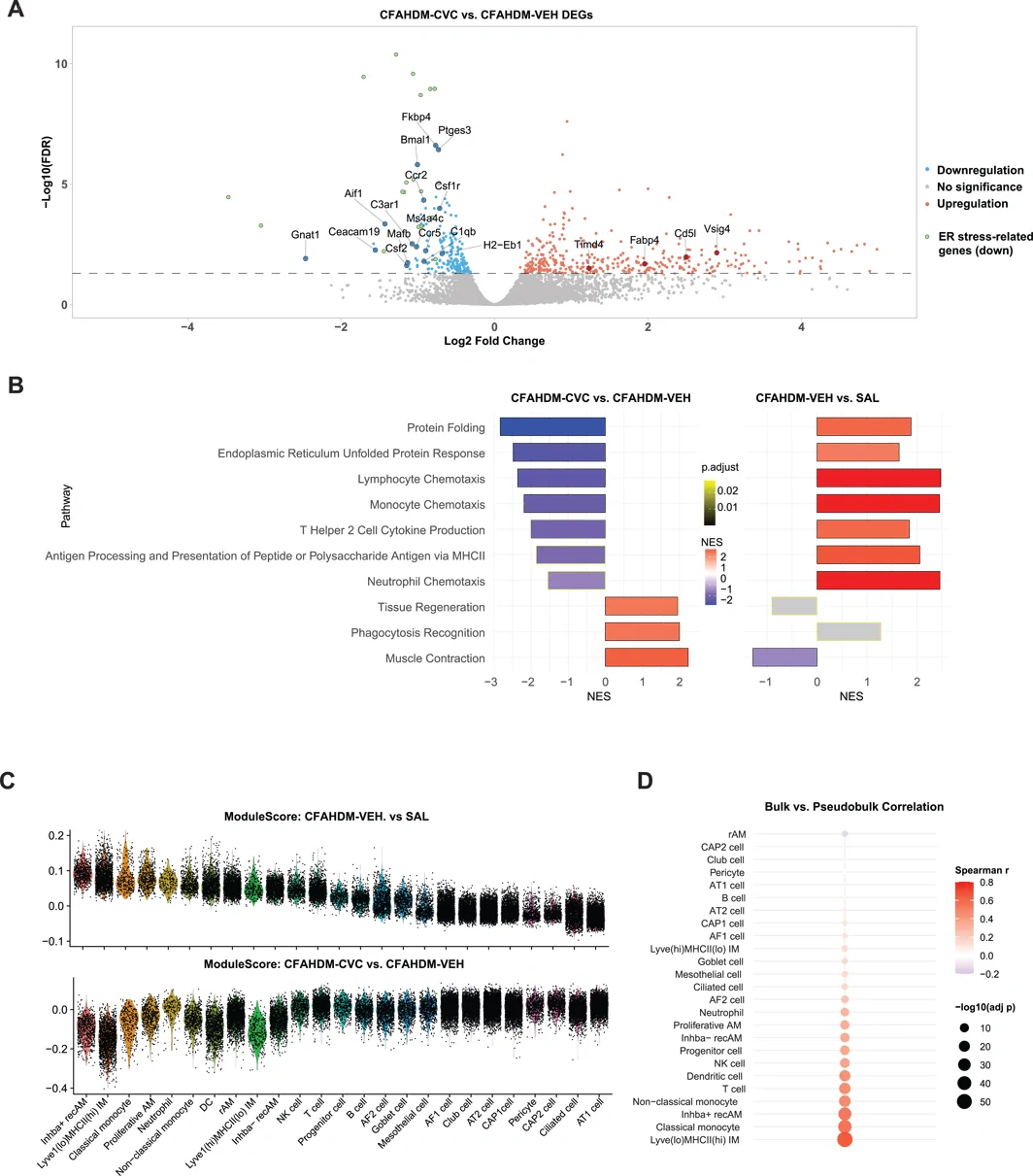
Transcriptomic and cell‐type‐specific analysis reveals CVC suppresses ER stress, chemotaxis, and inflammatory signaling in CFA‐HDM model. (A) Volcano plot showing differentially expressed genes (DEGs) between CFAHDM‐CVC and CFAHDM‐VEH groups (FDR < 0.05). Genes associated with ER stress are selectively downregulated (highlighted in blue), while inflammatory mediators such as *Ccl2*, *Tnf*, and *Fabp4* are downregulated. Pseudogenes were not presented. (B) Gene Set Enrichment Analysis (GSEA) comparing CFAHDM‐CVC versus CFAHDM‐VEH (left) and CFAHDM‐VEH versus SAL (right) groups. CVC suppressed pathways associated with ER stress, monocyte chemotaxis, and Th2 cytokine signaling. CFAHDM‐VEH mice exhibited upregulation of neutrophil and monocyte chemotaxis and phagocytic activity. Pathways with adjusted *p* < 0.05 are shown in colored bars; gray bars indicate adjusted *p* > 0.05. (C) Module scores for selected inflammatory and stress‐related pathways were projected onto scRNA‐seq data. CFAHDM‐VEH mice showed elevated stress and chemotaxis signals across multiple macrophage and monocyte subsets, which were reduced upon CVC treatment. (D) Correlation between bulk RNA‐seq signatures and pseudo‐bulk scRNA‐seq subsets. Myeloid subsets, including Lyve1^lo^MHCII^hi^ IMs and classical monocytes, showed strong positive correlation with bulk DEG signatures, highlighting their central role in disease and treatment response.

To further elucidate the molecular mechanisms of CCR2/CCR5 blockade, we performed bulk RNA‐seq analysis on lung tissue from SAL, CFA‐HDM‐VEH, and CFA‐HDM‐CVC groups. CVC treatment significantly attenuated the expression of genes associated with Endoplasmic Reticulum (ER) stress and the Unfolded Protein Response (UPR), such as heat shock protein (HSP)‐coding genes (green dots, Figure 6A). Genes linked to IM identity and differentiation, such as *Mafb*, *C1qb*, *Ccr2*, *Ccr5*, and *Csf1r* were downregulated, as were mediators of antigen presentation (*H2‐Eb1*), neutrophil function (*Gnat1*, *C3ar1*), and immune regulation (*Fkbp4*, *Ptges3*, *Bmal1*, *Aif1*, *Ceacam19*, *Ms4a4c*) (Figure 6A). In contrast, genes involved in tissue repair and lipid metabolism, which are key features of rAM function, were upregulated, including *Timd4*, *Fabp4*, *Cd5l*, and *Vsig4*.

Gene Ontology (GO) GSEA supported these findings, showing significant negative enrichment for pathways related to protein folding/ER stress, lymphocyte/monocyte/neutrophil chemotaxis, CCR chemokine receptor binding, Th2 cytokine production, and MHCII antigen presentation. Conversely, there was strong positive enrichment for processes involved in long‐chain fatty acid binding, tissue regeneration, phagocytic recognition, and other reparative programs (Figure 6B).

To integrate the bulk RNA‐seq data with scRNA‐seq data, we projected bulk‐derived gene signatures onto the single‐cell atlas. When upregulated DEGs from the CFA‐HDM versus SAL comparison were applied, the highest module scores were observed in CFA‐HDM‐induced AMs, Lyve1^lo^MHCII^hi^ IMs, and classical monocytes, suggesting these populations are major contributors to the disease‐associated transcriptional profile (Figure 6C). Similarly, when downregulated DEGs from the CFA‐HDM‐CVC versus CFA‐HDM‐VEH comparison were applied, these cell types showed the lowest module scores, indicating they were the primary targets of CVC‐mediated transcriptional reprogramming. Finally, correlation analysis between bulk DEGs and cell type‐specific pseudobulk DEGs (CVC vs. VEH) revealed the strongest positive correlation with Lyve1^lo^MHCII^hi^ IMs, while rAMs showed a negative association, highlighting opposing transcriptional trajectories between inflammatory IM subset and reparative rAMs in response to CVC treatment.

## 4 Discussion

Over the past decade, single cell and fate‐mapping studies have redefined the lung macrophage compartment, revealing that AMs and IMs are not uniform populations but comprise phenotypically and functionally distinct subsets with unique developmental origins and tissue niches [10, 11, 12, 13, 35]. However, how this heterogeneity contributes to asthma pathogenesis remains poorly defined, with conflicting reports suggesting both protective and inflammatory roles for different macrophage populations [7, 16, 19, 20, 23, 26]. Here, we show that experimental severe asthma is characterised by a profound loss of rAMs, accompanied by the emergence of monocyte‐derived recAMs, with minimal contribution from the self‐proliferative AMs. We further identified two previously reported IM clusters of monocyte origin [10] and annotated their distinct functions: the Lyve1^lo^MHCII^hi^ population exhibited pro‐inflammatory and antigen‐presenting features, while the Lyve1^hi^MHCII^lo^ subset displayed immunosuppressive and wound‐healing properties. Notably, the Lyve1^hi^MHCII^lo^ IMs remained relatively stable during disease progression, whereas the Lyve1^lo^MHCII^hi^ subset became overpopulated and dominated both the bronchoalveolar space and lung parenchyma. These pro‐inflammatory Lyve1^lo^MHCII^hi^ IMs expressed high levels of *Ccr2* and *Ccr5*, and dual antagonism with CVC selectively blocked this subset without affecting the Lyve1^hi^MHCII^lo^ counterparts. This intervention led to reduced T cell infiltration, decreased neutrophil and eosinophil recruitment, improved lung histopathology and attenuated methacholine‐induced AHR.

In patients with severe asthma from the U‐BIOPRED cohort, reduced macrophage numbers have been observed in sputum cell samples, with lower rAM signature enrichment linked to worse lung function [36]. Our in silico analysis provided further context to this observation: while rAM‐associated genes were consistently decreased, IM‐ and monocyte‐related signatures were increased. A similar phenomenon occurs in allergic asthma [24] and was recapitulated in our CFA‐HDM mouse model, where flow cytometry identified near complete loss of Siglec‐F^bright^ rAMs in BAL and their replacement by Siglec‐F^high^ CD11b^dim^ recAM populations. These recAMs phenotypically resembled both rAMs and IMs in their surface protein expression and transcriptomic profiles, indicative of an intermediate differentiation state. As Siglec‐F is a defining marker of terminally differentiated rAMs, which is acquired during development from fetal monocytes or hematopoietic progenitors with concurrent CD11b downregulation and CD11c upregulation [37, 38], the Siglec‐F^high^ CD11b^dim^ recAMs likely represents an immature or transitional AM state [35, 37]. Supporting these findings, a recent human single‐cell study similarly demonstrated a reduction in rAM abundance with a concomitant increase in monocyte‐derived, macrophage‐like cells (MCs) in endobronchial brush samples from patients with allergic asthma [39]. Moreover, following bronchoscopic segmental allergen challenge (SAC), the airway space is overpopulated with *CCR2* ^hi^‐MCs and their descendants along the differentiation trajectory toward macrophages, including subsets enriched for pathways of antigen processing and presentation, and genes for tissue remodeling (*MMP‐9* and *MMP‐12*) [39]. Consistently, during asthma exacerbations, BAL accumulation of monocytes and *MAF*‐regulated mo‐Macs with high expression of Human Leukocyte Antigen (HLA) genes has also been reported [40]. As human monocyte‐derived alveolar macrophages (mo‐AMs) share substantial transcriptional overlap with IM populations, these findings collectively suggest a shift away from homeostatic rAM programs toward monocyte‐derived macrophage states in severe asthma, as previously observed in COPD smokers [41].

The presence of ER stress in our CFA‐HDM model aligns with previous clinical observations in sputum and bronchial biopsies from patient with severe asthma, which further demonstrated ER stress is associated with clinical severity and inflammatory phenotypes [42]. In macrophages, ER stress has been linked to pro‐inflammatory macrophage polarisation [43], supporting the macrophage subset shift observed in our model. Among the two recAM clusters, the Inhba^+^ subset was particularly enriched in pathways related to proteolysis, glycolysis, cytokine production, and tissue remodeling, suggesting a potential role in asthma pathogenesis. *Inhba* encodes the Inhibin beta A subunit, a regulator of macrophage polarisation [44], and marks a macrophage population linked to cigarette smoking‐induced emphysema and lung carcinoma progression [45, 46], although its role in asthma has not been previously defined. In addition to the emergence of the recAMs, residual rAMs in the CFA‐HDM exposed mice also appeared to be functionally altered based on the transcriptomic analysis. rAMs are typically immunosuppressive [23, 24, 47], but this phenotype can be reversed by pro‐inflammatory cytokines such as GM‐CSF [48]. Thus the allergen‐rich, cytokine‐ and oxidative stress‐laden environment of severe asthma may drive rAMs toward a pro‐inflammatory state, triggering intrinsic and extrinsic cell death pathways [49, 50] and contributing to their depletion.

Compared to AMs, IMs are less extensively studied and have classically been viewed as protective and anti‐inflammatory [7, 8, 10, 19]. In mice, IMs constitutively produce IL‐10. Upon LPS and OVA antigen challenge, they have been shown to attenuate LPS‐induced maturation and migration of antigen‐loaded DCs, thereby preventing the induction of a Th2 response [8]. These effects are both IL‐10 and TLR4 dependent [7, 8]. Similarly, TLR9 activation with unmethylated CpG DNA expands IMs and protects against HDM‐induced airway inflammation through IL‐10 production in a CCR2‐independent manner [19]. However, pro‐inflammatory roles for the IMs have also been reported. For instance, CD11c^+^ IMs with high MHCII expression can capture and present inhaled antigen to drive Th2 inflammation [25, 51, 52], while IL‐9 activated IMs produce excessive Arg1 and CCL5 to promote allergic inflammation [53]. These contrasting findings likely reflect functional heterogeneity within the IM compartment. Recent research has helped clarify this dichotomy by showing that IL‐10‐producing, wound‐healing functions are largely restricted to the Lyve1^hi^MHCII^lo^ IM subset, depletion of which exacerbated bleomycin‐induced pulmonary fibrosis [10]. In contrast, the Lyve1^lo^MHCII^hi^ subset likely corresponds to the previously described CD11c^+^MHCII^hi^ pro‐inflammatory IMs. Our study confirms this relationship: the Lyve1^lo^MHCII^hi^ IMs identified by scRNA‐seq showed high CD11c and MHCII protein expression and were markedly expanded in the CFA‐HDM‐exposed lungs. These cells displayed a robust pro‐inflammatory profile, promoting the recruitment and activation of T cells, neutrophils, eosinophils, and monocytes. They also appeared to reprogram rAM (e.g., via Plau—Plaur interaction) and may possibly contribute to their disappearance under disease conditions. Targeting this population with CVC was sufficient to disrupt the intercellular network, lower airway inflammation and partially restore lung homeostasis and lung function. These findings thus expand our understanding of the dualistic roles of IM subsets and their distinct contributions to asthma pathogenesis [28, 42, 43, 54, 55].

Beyond asthma, recent single‐cell studies in pulmonary arterial hypertension (PAH) similarly identified Lyve1^lo^MHCII^hi^ IMs as key inflammatory mediators of vascular fibrosis, with myeloid‐specific deletion of *Ccr2* attenuating this IM‐driven pathogenic program [56]. Notably, dual CCR2/CCR5 blockade was more effective than single‐receptor inhibition in preventing the emergence of these pathogenic macrophages and interrupting their crosstalk to smooth muscle cells that drives vascular remodeling [57]. Consistently, CVC reduced fibrosis in mouse models of liver and kidney disease by targeting monocyte‐derived macrophages [28], and in patients with HIV, treatment was associated with reduced monocyte activation markers [54] and fibrotic biomarkers [55].

While our study offers novel insights into macrophage heterogeneity in severe asthma, several limitations should be considered. First, the 10X Genomics platform has limited sensitivity for granulocyte detection, leading to underrepresentation of neutrophils and an absence of eosinophils in our scRNA‐seq dataset. The scRNA‐seq experiments were also primarily designed as discovery‐based analyses and limited in statistical power. These limitations were partially mitigated through parallel flow cytometry profiling, which confirmed the expansion of these populations in CFA‐HDM mice and reduction in the IMs following CVC treatment. Second, we focused on a single model of severe steroid‐resistant asthma and administered CVC in a prophylactic manner. Although this approach demonstrates proof‐of‐concept efficacy, future studies should investigate therapeutic dosing following HDM challenge and employ genetic models with macrophage‐specific deletion of *Ccr2*/*Ccr5* to strengthen cell‐type specificity. Lastly, while our transcriptomic analysis of the sputum samples provided supportive evidence, human lung macrophage subsets remain less well defined than those in mice and warrant further investigation, particularly after sex‐stratification. Our preliminary sex‐stratified analyses of the U‐BIOPRED and Australian cohorts did not identify significant differences in IM populations between males and females (not shown); however, caution needs to be taken given their reliance on in silico cell‐type estimation.

In summary, our study highlights a previously underappreciated level of functional heterogeneity within the lung macrophage compartment, with direct implications for targeted therapy in asthma. The Lyve1^lo^MHCII^hi^ IMs subset emerged as a central driver of airway inflammation, displaying potent pro‐inflammatory and antigen‐presenting programs and uniquely expressing CCR2 and CCR5. Preferential reduction of this subset with CVC, a clinically tested dual CCR2/CCR5 antagonist with a favorable safety profile [58] effectively attenuated airway inflammation, preserved resident macrophage populations and improved lung function. These findings position CCR2/CCR5 blockade as a promising macrophage‐directed therapeutic strategy and provide a mechanistic framework for future clinical studies in severe, steroid‐resistant asthma.

## Author Contributions

**Hao Wang:** conceptualization, writing – original draft, funding acquisition, investigation, methodology, validation, visualization, writing – review and editing, data curation, supervision, resources, project administration, formal analysis, software. **Nok Him Fung:** investigation, data curation, methodology, validation, visualization, writing – review and editing, formal analysis. **Christian Aloe:** investigation, methodology, validation, visualization, writing – review and editing, formal analysis. **Nazanin Zounemat Kermani:** investigation, data curation, methodology, validation, visualization, writing – review and editing, formal analysis. **Ian M. Adcock:** investigation, data curation, supervision, methodology, validation, visualization, writing – review and editing, formal analysis. **Stavros Selemidis:** investigation, supervision, methodology, validation, visualization, writing – review and editing, formal analysis. **Ross Vlahos:** investigation, supervision, methodology, validation, visualization, writing – review and editing, formal analysis. **Jonathan McQualter:** funding acquisition, writing – original draft, investigation, supervision, methodology, validation, visualization, writing – review and editing, formal analysis. **Steven Bozinovski:** conceptualization, investigation, writing – original draft, funding acquisition, data curation, supervision, resources, project administration, formal analysis, software, methodology, validation, visualization, writing – review and editing.

## Funding

This study was funded by a grant from CASS Foundation (#10466).

## Conflicts of Interest

The authors declare no conflicts of interest.

## Supporting information


Supporting Information S1

## Data Availability

All sequencing data generated in this study have been deposited in the EMBL Nucleotide Archive under accession number PRJEB112062.
