# Supplementary material for: CCR2/CCR5 Signaling Defines a Pathogenic Interstitial Macrophage Subset That Drives Severe Asthma
Source: Clin Transl Allergy. 2026 Sep 12;16(9):e70201. doi: 10.1002/clt2.70201 (PMC13570780; doi:10.1002/clt2.70201)
Supplement: Supplementary file 1 [file CLT2-16-e70201-s001.docx]

**Supporting Information for**

CCR2/CCR5 signaling defines a pathogenic interstitial macrophage subset that drives severe asthma.

**Authors:** Hao Wang ^1, 2^*, Nok Him Fung ^1, 2^, Christian Aloe ^1, 2^, Nazanin Zounemat Kermani ^3^, Ian M Adcock ^4^, Stavros Selemidis^1, 2^, Ross Vlahos ^1, 2^, Jonathan McQualter ^1, 2^, Steven Bozinovski ^1, 2^*

**Affiliations:**

^1^ RMIT University, School of Health and Biomedical Sciences, Victoria, Australia

^2^ Centre for Respiratory Science & Health, Victoria, Australia

^3^ Data Science Institute, Imperial College London, London, UK

^4^ National Heart and Lung Institute, Imperial College London, London, UK, on behalf of the U-BIOPRED consortia

*Joint corresponding authors:

Dr Hao Wang, School of Health, and Biomedical Sciences, RMIT University, PO Box 71 Bundoora VIC 3083 Australia; Phone: +61 3 9925 7243; Email: [hao.wang@rmit.edu.au](mailto:hao.wang@rmit.edu.au)

Prof Steven Bozinovski, School of Health and Biomedical Sciences, RMIT University, PO Box 71 Bundoora VIC 3083 Australia; Phone: +61 3 9925 6674; Email: [steven.bozinovski@rmit.edu.au](mailto:steven.bozinovski@rmit.edu.au)

**Supplementary Materials and Methods**

**Lung function tests**

Respiratory mechanics were measured *in vivo* using a flexiVent system (flexiVent FX1; SCIREQ Scientific Respiratory Equipment, Montreal, Quebec, Canada). Briefly, mice were anesthetised with ketamine (125 mg/kg) and xylazine (25 mg/kg) before being tracheostomised and cannulated. Mice were then connected to the flexiVent, and baseline compliance, elastance and pressure-volume (PV) loop parameters were measured. This was followed by the stepwise delivery of nebulised saline and increasing doses of methacholine (Mch, 3-50 mg/mL) to the lungs to assess respiratory resistance.

**BAL and tissue collection**

Following lung function tests, mice were euthanised via pentobarbital overdose. Bronchoalveolar lavage (BAL) was then performed to collect BAL cells for flow cytometry, and total cell numbers were quantified using a hemocytometer, and total cells recovered were counted using a hemocytometer. Lungs were perfused with ice-cold PBS to remove blood. The superior lung lobe was excised for flow cytometric analysis, while the left lobe was fixed in 10% neutral-buffered formalin (NBF) for histological evaluation. In the intervention study, the remaining lung lobes from SAL (n = 2), CFAHDM-VEH (n = 4), and CFAHDM-CVC (n = 4) groups were flash-frozen for bulk RNA sequencing.

**Flow cytometry**

The superior lung lobe was finely minced and digested in Liberase TM (Sigma-Aldrich, US) at 37°C with constant shaking. Single cell suspensions were prepared by passing the digested tissue through a 21-gauge needle and then a 40-μm cell strainer. This cell suspension was centrifuged and treated with ammonium-chloride-potassium red blood cell lysis buffer. After blocking with CD16/CD32 antibody, both BAL cells and lung cells were stained with a Fixable Viability Dye eFluor™ 780 (Life Technologies, US) before a myeloid cell antibody cocktail (**Table S1**), or a CCR2/CCR5 antibody cocktail (**Table S2**). Following staining, cells were fixed with an IC Fixation Buffer (Thermo Fisher Scientific, US) before being analysed on a BD FACSAria flow cytometer (BD Biosciences, US). All antibodies and reagents were obtained from Thermo Fisher Scientific unless otherwise stated. Data were analysed with FlowJo v10.7.2 software (BD Biosciences, US).

**Lung histology**

The NBF-fixed lung lobes were processed, paraffin-embedded, and sectioned at 4 μm. Sections were stained with hematoxylin and eosin (H&E) for assessment of lung inflammation. Stained whole-lung sections were scanned using a VS-120 Olympus slide scanner and Olympus cellSens Dimension software (Olympus Life Science, US). Lung inflammation was scored in a blinded manner on a scale of 0 to 3 (none to severe) in peribronchial, perivascular, and interstitial/alveolar regions individually based on the degree of inflammatory cell infiltration.

**Single-cell RNA library preparation and sequencing**

A separate cohort of mice across four groups (n = 3 per group): SAL, CFAHDM, CFAHDM-VEH, and CFAHDM-CVC were used for scRNA-seq analysis. Mice were euthanised by pentobarbital overdose. Whole lungs were collected and immediately processed for fixation and dissociation following the Tissue Fixation & Dissociation for Chromium Fixed RNA Profiling protocol (10x Genomics, US). The dissociated cells were stored at -80 $^{\circ}$C prior to shipping to Australian Genome Research Facility (AGRF, Melbourne, Australia) for probe hybridisation, Gel bead-in-Emulsion (GEM) generation and barcoding, and library preparation using the Chromium Fixed RNA Profiling Reagent Kits (10x Genomics, US). Paired-end 150-bp sequencing was performed on the NovaSeq X platform.

**Single-cell RNA-seq data processing and bioinformatic analysis**

Raw FASTQ files were demultiplexed and mapped to the mouse reference genome (mm10) using the 10x Genomics CellRanger v7.0 ‘cellranger multi’ pipeline in R 4.3.3. Unique molecular identifier (UMI) counts were quantified, and low-quality cells were removed. The processed data were imported and merged in the *Seurat* R package (v4.4.0) for downstream analysis. Gene expression features detected in at least 3 cells, with a number of features per cell between 200 and 6,000, were retained. Cells with a total RNA count greater than 10,000, mitochondrial gene expression exceeding 10% of the total counts, or hemoglobin gene expression over 1% were filtered out. Doublets were removed using the *DoubletFinder* package (v2.0.3). Gene expression data from the high-quality cells (37,211 cells) were normalised and scaled using default parameters, followed by principal component analysis (PCA) and dimensionality reduction.

*Harmony* package (v1.2.0) was used to integrate all cells from different treatment groups and correct for batch effects and treatment variables. The ‘FindNeighbors’ function (with the top 20 dimensions) and ‘FindClusters’ function (with a resolution of 0.6) were used for unbiased clustering, and clusters were visualised using Uniform Manifold Approximation and Projection (UMAP). Marker genes for each cluster were identified using the ‘FindAllMarkers’ function, and cell type annotation was performed manually based on previously reported marker genes ^1,2^. A total of 20 clusters, comprising 36,824 cells, were annotated.

To further resolve the myeloid lineage, where neutrophils, monocytes, and macrophage subsets (8,389 cells) lacked clear distinction, sub-clustering was performed using the ‘FindNeighbors’ function (with the top 20 dimensions) and ‘FindClusters’ function (with a resolution of 0.5). This revealed 8 clusters of myeloid cells (7,276 cells) based on marker genes identified by the ‘FindAllMarkers’ function. These subclusters were then re-annotated in the main *Seurat* object, resulting in 36,238 cells representing 25 cell types visualised in a UMAP. Of these, 16,240 cells were derived from SAL-treated mice and 20,584 from the CFA-HDM groups. For group-wise comparative analysis, cells were downsampled to match the group with the lowest cell count, thereby ensuring that an equal number of cells were used across groups. The *ClusterGVis* (v0.1.2) package was used to visualise the top 100 marker genes for each cluster in a heatmap and to perform enrichment analysis on these marker genes using the GO database.

**Cell-cell communication analysis**

Cell-cell communication analysis was conducted on all identified cells using the Ligand-Receptor Interactions Analysis (*LIANA*) package (v 0.1.14). First, ligand-receptor pairs were predicted based on known interactions curated from several databases such as *CellPhoneDB*, *OmniPath*, and others integrated within *LIANA*. These interactions were further ranked by interaction scores and statistical significance. We focused on the Lyve1^lo^MHCII^hi^ IMs and visualised this population as both signal senders and receivers.

**Cell trajectory analysis**

Cell trajectory analysis was performed on the macrophage and monocyte subsets using the *Monocle* package (v2.30.1). This analysis reconstructs cellular developmental trajectories based on gene expression profiles, enabling the inference of differentiation pathways and dynamic gene expression changes over pseudo-time. The pre-processed data from the Seurat object, containing the macrophage and monocyte subsets, were first imported into Monocle 2. Dimensionality reduction was performed using the DDRTree algorithm to visualise the cell trajectories in a two-dimensional UMAP. Pseudo-time ordering was applied to infer the progression of cells along distinct differentiation paths. Root and branch points within the trajectory were identified to delineate key transitional stages in macrophage and monocyte maturation. To further improve the robustness of the analysis, the *CytoTRACE* package (v0.3.3) was independently applied. *CytoTRACE* calculates the differentiation state of each cell by analysing transcriptional programs related to stemness or differentiation. The *CytoTRACE* results were integrated into the Monocle object, and the *CytoTRACE* scores were visualised on the cell trajectory plot as previously described ^3^.

***In silico* analysis of U-BIOPRED sputum transcriptome**

U-BIOPRED sputum microarray data (GSE76262) was downloaded from Gene Expression Omnibus (GEO), and differential gene expression analysis was performed using the *Limma* package (v3.58.1). Human single-cell reference dataset (LungMAP_HumanLung_CellRef.v1.1.h5ad) was downloaded from LungMap portal and imported into *Seurat* (v4.4.0) for downstream analysis. Bulk gene signatures consisting of differentially expressed genes (DEG, defined as false discovery rate or FDR < 0.05 and |logFC| > 0.5) upregulated or downregulated in the severe asthma vs. healthy controls comparison were intersected with the feature space of the single-cell dataset. Cell-level enrichment scores of the DEGs were computed using *Seurat*’s AddModuleScore function. These scores were appended to cell metadata and visualised across annotated cell populations using DimPlot. The ‘gsva’ function from the *GSVA* R package (v1.50.5) was used with default parameters to compute enrichment scores (ES). The Gene Ontology (GO) database was utilised as a reference for the functional gene sets. Macrophage/monocyte-related genes established by LugMap were visualised in a heatmap using the *pheatmap* package (v1.0.12). Hierarchical clustering was applied to rows (genes) to group similar expression patterns.

**Cellular deconvolution of lung transcriptome from asthma cohorts**

The lung single-cell reference dataset was downloaded from the Human Lung Cell Atlas (HLCA) portal and imported into Python for processing. An airway-specific single-cell reference matrix was constructed following the established HLCA deconvolution pipeline (https://github.com/LungCellAtlas/HLCA). Cellular deconvolution of epithelial brushing and bronchial biopsy transcriptomes from three independent severe asthma cohorts: U-BIOPRED, Severe Asthma Research Program (SARP) (GSE63142), and an Australian cohort (GSE143303) was then performed using this airway single-cell reference in CIBERSORTx as described earlier ^4^. CIBERSORTx-estimated cell fractions were visualised using *ggplot2* package (v3.4.4) in R studio.

Asthma severity was classified according to the original cohort definitions. In U-BIOPRED cohort, participants with severe asthma with asthma had uncontrolled symptoms defined according to Global Initiative for Asthma (GINA) guidelines and/or frequent exacerbations despite high-dose inhaled corticosteroids, whereas participants with mild/moderate asthma were nonsmokers for at least 12 months, had a smoking history less than 5 pack-year and had controlled or partially controlled asthma symptoms, according to GINA criteria while receiving <500 µg fluticasone propionate/day or equivalent. In the SARP cohort (GSE63142), participants were classified as healthy controls, non-severe asthma, or severe asthma according to the original study classification, with severe asthma defined according to the American Thoracic Society 2000 definition.

**Gene set enrichment analysis of *Ccr2* and *Ccr5***

The human and mouse lung single-cell reference datasets were downloaded from the LungMap portal and imported into *Seurat* (v4.4.0). *Ccr2* and *Ccr5* were combined as a gene set, and their cell-level enrichment scores were computed using *Seurat*’s AddModuleScore function. These scores were appended to cell metadata and visualised across annotated cell populations using DimPlot. The same analytical workflow was applied to our in-house scRNA-seq dataset from SAL-treated or CFAHDM-exposed mice. For the U‑BIOPRED sputum transcriptome, enrichment of the CCR2-CCR5 gene set was calculated using the gsva function from the *GSVA* package, with ES computed at single-sample level.

**Bulk RNA library preparation and sequencing**

Total RNA was isolated from crushed frozen lung tissue using a RNeasy Plus kit (Qiagen, Germany) and shipped to the Australian Genome Research Facility (AGRF, Melbourne, Australia) for sequencing. RNA purity and integrity were assessed using an Agilent TapeStation System (Agilent Technologies, Santa Clara, California, US) prior to library construction with the Illumina Stranded mRNA Prep (Illumina, San Diego, California, US). Twenty million 150-bp paired-end reads were performed on the Illumina NovaSeq X plus platform, and primary sequence data was then generated with the Illumina DRAGEN BCL Convert 07.021.645.4.0.3 pipeline. The raw reads were analysed in FastQC and trimmed with TrimGalore-0.6.7 to remove low-quality reads and adaptor sequences. The *STAR* aligner (v 2.7.4a) was used to map reads against the Mus musculus genome (Build version mm39) and featureCounts from the *Subread* package (v2.0.3) was used to generate the raw gene counts. Low gene counts were removed in *edgeR* (v 3.38.4), followed by the trimmed mean of M-values (TMM) normalisation and differential gene analysis. DEGs were defined as having a false discovery rate (FDR) < 0.05.

**Bulk RNA-seq data analysis**

Gene Set Enrichment Analysis (GSEA) was performed on pre-ranked gene lists derived from differential expression statistics using GO biological process gene sets. Enrichment testing and visualisation were conducted using the *clusterProfiler* (v4.2.2), *enrichplot* (v1.14.2), and *ggplot2* (v3.4.4) packages. To map bulk transcriptomic alterations to specific cellular compartments, we integrated bulk and single-cell RNA-seq datasets. Bulk gene signatures consisting of DEGs upregulated in the CFAHDM-VEH vs. SAL comparison and downregulated in the CFAHDM-CVC vs. CFAHDM-VEH comparison were intersected with the feature space of the single-cell dataset. Cell-level enrichment scores were computed using *Seurat*’s AddModuleScore function, with downregulated signatures manually scaled to negative values to indicate inverse regulation. These scores were appended to cell metadata and visualised across annotated cell populations using violin plots. For pseudobulk analyses, raw single-cell expression counts were aggregated at the sample level by cell type using the ‘AggregateExpression’ function to allow group-level comparisons. Differential expression at the pseudobulk level was performed using *edgeR* and *limma* (TMM normalisation followed by voom transformation and linear modelling), and concordance with bulk RNA-seq results was assessed by calculating Spearman’s rank correlation coefficients between matched gene-wise log_2_ fold-changes. Multiple testing correction was applied using the *Benjamini-Hochberg* method. The degree of concordance was visualised using *ggplot2* package.

**Data analysis**

All data were statistically analysed using GraphPad Prism 9.0 (GraphPad, US) or R studio. Data are presented as the mean ± SEM or median with interquartile range (IQR). Where detailed and appropriate, one-way analysis of variance or Kruskal-Wallis test was performed. p < 0.05 is considered to be statistically significant.

**Supplementary Tables**

**Table S1. Antibodies used for flow cytometric analysis of lung myeloid cells**

| **Antibody Specificity** | **Conjugate** | **Clone** | **Dilution** | **Supplier** |
| --- | --- | --- | --- | --- |
| CD11b | PE Dazzle 594 | M1/70 | 1:400 | Biolegend |
| CD11c | BV650 | N418 | 1:250 | Biolegend |
| CD45 | AlexaFluor700 | 30-F11 | 1:100 | Biolegend |
| CD64 | PE Cy7 | X54-5/71 | 1:150 | Biolegend |
| CD49b | BV711 | HMα2 | 1:100 | BD |
| Ly6C | AlexFluor488 | HK1.4 | 1:300 | Biolegend |
| Ly6G | BV785 | 1A8 | 1:250 | Biolegend |
| MerTk | APC | 2B10C42 | 1:200 | Biolegend |
| MHCII | PerCp Cy5.5 | M5/114.15.2 | 1:150 | Biolegend |
| SiglecF | PE | E50-2440 | 1:300 | BD Pharmingen |
| Lyve1 | eFluor450 | ALY7 | 1:200 | Life Technologies |
| MHCII | PE-Cyanine5 | I-A/I-E | 1:200 | Biolegend |

**Table S2. Antibodies used for flow cytometric analysis of CCR2 and CCR5 protein expression**

| **Antibody Specificity** | **Conjugate** | **Clone** | **Dilution** | **Supplier** |
| --- | --- | --- | --- | --- |
| CD11b | PE Dazzle 594 | M1/70 | 1:400 | Biolegend |
| CD11c | BV650 | N418 | 1:250 | Biolegend |
| CD45 | AlexaFluor700 | 30-F11 | 1:100 | Biolegend |
| CD64 | PE Cy7 | X54-5/71 | 1:150 | Biolegend |
| CD49b | BV711 | HMα2 | 1:100 | BD |
| CCR5 | AlexFluor488 | HM-CCR5 | 1:200 | Biolegend |
| Ly6G | BV785 | 1A8 | 1:250 | Biolegend |
| MerTk | APC | 2B10C42 | 1:200 | Biolegend |
| MHCII | PerCp Cy5.5 | M5/114.15.2 | 1:150 | Biolegend |
| SiglecF | PE | E50-2440 | 1:300 | BD Pharmingen |
| Lyve1 | eFluor450 | ALY7 | 1:200 | Life Technologies |
| CCR2 | PE-Cyanine5 | SA203G11 | 1:200 | Biolegend |

**Supplementary Figures**

Figure S1. Single-cell expression of dendritic/macrophage lineage genes across lung immune populations.

Violin plots show the expression of macrophage lineage genes, including *Ccr2*, *Ccr5*, *Mafb*, and *Mertk*, *Fcgr1*, *Adgre1*, *Ms4a7*, and *Lyz2*, along with conventional dendritic cell (cDC) markers *Zbtb46*, *Flt3*, *Xcr1*, and *Clec9a*, as well as the dendritic lineage transcription factors *Irf4* and *Irf8*. Expression is shown across annotated lung immune cell subsets. Each dot represents an individual cell.

Figure S2. Cell type alterations in CFA-HDM lungs identified by scRNA-seq and flow cytometry.

(**A**) scRNA-seq was performed on total lung cells from three mice per experimental group (n = 3): SAL (blue), CFAHDM (orange). Cell type fractions were calculated after downsampling to equal cell numbers per group. Data are presented as median with interquartile range (IQR). To validate the macrophage subset shifts identified by scRNA-seq, flow cytometry was conducted on BAL cells and post-lavage lung tissue. (**B-C**) Representative gating strategies for (**B**) BAL cells and (**C**) lung tissue macrophages are shown.

Figure S3. Marker gene expression and pseudotime trajectory profiling of macrophage subsets.

(**A**) Dot plot showing the expression of representative feature genes (*Il10*, *Tnf*) across key macrophage subsets identified by scRNA-seq. Dot size indicates the proportion of cells expressing the gene, while color intensity represents average scaled expression. (**B**) Heatmap depicting gene expression dynamics across pseudotime clusters derived from trajectory analysis. Cells are ordered along the trajectory with branch identities and cell type annotations shown at the top. Cell fate 1 is IM and Cell fate 2 is recAM. Three distinct gene expression modules (Clusters 1-3) were identified, highlighting temporal transcriptional programs associated with monocyte-to-macrophage differentiation and inflammatory activation.

Figure S4. Differential expression of AM-, IM- and cMon-associated gene signatures in sputum cells across asthma severity.

(**A**) Violin plots showing GSVA enrichment scores (ES) of AMs, IMs, classical monocytes, and non-classical monocytes across disease states. IM and classical monocyte scores were significantly increased in severe asthma, while CCR2-CCR5 showed a trend toward increase. (**B**) Violin plots showing enrichment of selected gene ontology terms relevant to monocyte and macrophage function. Severe asthma patients showed increased enrichment of monocyte chemotaxis, monocyte differentiation, and CCR receptor binding. Data are presented as median with interquartile range (IQR). * p< 0.05, ** p< 0.01, *** p< 0.001, **** p< 0.0001 by Kruskal–Wallis test with Dunn’s multiple comparisons.

**Figure S5. Cellular deconvolution of epithelial brushing and bronchial biopsy transcriptomes by CIBERSORTx.**

Cellular deconvolution of epithelial brushing and bronchial biopsy transcriptomes from three independent severe asthma cohorts: U-BIOPRED, SARP (GSE63142), and an Australian cohort (GSE143303), was performed using an established airway single-cell reference and analytical pipeline from the Human Lung Cell Atlas (HLCA). Data are presented as median with interquartile range (IQR).

 Figure S6. Gene set enrichment of *Ccr2* and *Ccr5* in the LungMAP single-cell atlases and in SAL-treated or CFAHDM-exposed mice.

Cell-level enrichment of *Ccr2* and *Ccr5* as a gene set (calculated by *Seurat* AddModuleScore) are visualised on UMAPs of (**A**) the human LungMAP atlas, (**B**) the mouse LungMAP atlas, and our in-house scRNA-seq dataset from (**C**) SAL-treated and (**D**) CFAHDM-exposed mice.

Figure S7. CCR2 and CCR5 proteins are co-expressed in Lyve1^lo^MHCII^hi^ IMs in the mouse lung assessed by flow cytometry.

To assess the CCR2 and CCR5 protein expression in the Lyve1^lo^MHCII^hi^ IM subset, flow cytometry was conducted on BAL cells and post-lavage lung tissue from 4 SAL mice and 8 CFAHDM mice. (A) Representative gating strategies for BAL cells and lung tissue macrophages are shown, with overlaid plots illustrating CCR2 and CCR5 expression across immune cell subsets. Double-positive cells were predominantly Lyve1^lo^MHCII^hi^ IMs. (B) Quantification of median fluorescence intensity (MFI) of CCR2 and CCR5 expression across the immune cell subsets identified in (A).

**Figure S8.** **CCR2/CCR5 antagonism lowers Th1/Th2 responses and inflammatory gene expression.**

scRNA-seq data were downsampled to equal cell numbers per group across SAL, CFAHDM-VEH, and CFAHDM-CVC groups. (**A**) Cell type fraction bar graphs show Th1 (*Ifng*, *Tbx21*, *Cxcr3*) and Th2 (*Il4*, *Il5*, *Il13*) subsets in T cells were increased in CFAHDM-VEH, and reduced following CVC. Data are presented as median with interquartile range (IQR). (**B**) Dot plots show scaled average expression (colour) and proportion of expressing cells (size) for selected immune-related genes across the groups. Three gene expression panels highlight myeloid-associated chemokine signalling (left), T helper/Treg-associated transcriptional programs (middle), as well as macrophage subset markers and inflammatory mediators (right), which were altered in CFA-HDM-VEH and partially prevented by CVC treatment.

**Reference**

1.	Guo M, Morley MP, Jiang C, et al. Guided construction of single cell reference for human and mouse lung. *Nat Commun.* 2023;14(1):4566.

2.	Spix B, Butz ES, Chen CC, et al. Lung emphysema and impaired macrophage elastase clearance in mucolipin 3 deficient mice. *Nat Commun.* 2022;13(1):318.

3.	Cheng S, Li Z, Gao R, et al. A pan-cancer single-cell transcriptional atlas of tumor infiltrating myeloid cells. *Cell.* 2021;184(3):792-809 e723.

4.	Sikkema L, Ramirez-Suastegui C, Strobl DC, et al. An integrated cell atlas of the lung in health and disease. *Nat Med.* 2023;29(6):1563-1577.
